# The Effects of War and Trauma on Attitudes Toward Restorative Justice for Sexual Offenses

**DOI:** 10.1192/j.eurpsy.2025.2427

**Published:** 2025-08-26

**Authors:** I. Peleg Koriat, D. Weimann Saks

**Affiliations:** 1 Criminology; 2 Communication, Max Stern Yezreel Valley College, Yezreel Valley, Israel

## Abstract

**Introduction:**

On October 7, 2023, a Hamas terror attack targeted both Jewish and Arab Israelis. The unprecedented scale of the event resulted in over 1,400 deaths and numerous kidnappings, leading to the ongoing Hamas-Israel war. The attacks also exposed a wide range of sexual violence, which was broadcasted or witnessed by family members (Association of Rape Crisis Centers in Israel, 2024; UN SRSG-SVC, 2024). This event has exacerbated collective trauma and anxiety, influencing various societal issues, including public attitudes toward justice, particularly sexual offenses (Feingold et al., 2024).

**Objectives:**

This study aims to explore how exposure to a war characterized by violent crimes affects public support for restorative justice (RJ) processes, particularly in sexual offenses, and whether the collective trauma has led to a decline in the belief that sexual offenders can be rehabilitated.

**Methods:**

The research surveyed 339 Israeli participants at two points: before the war and during its early weeks. The study employed quantitative analyses to measure attitudes toward offender rehabilitation and support for RJ. A paired t-test was conducted to assess changes over time, and correlation analyses examined the relationship between the perceived malleability of offenders and support for RJ.

**Results:**

The findings revealed a significant decline during the war in the belief that sexual offenders can be rehabilitated (*t*(51) = 2.67, *p* < .01). This belief was correlated to reduced support for RJ (rp = .45, *p* < .001). The exposure to sexual violence during the war, combined with widespread testimonies highlighting its brutality, likely contributed to a shift in public attitudes. The attacks served to instill terror and dehumanize the victims, reinforcing the public’s resistance to perceived “lenient” justice alternatives such as RJ.

**Conclusions:**

In addition to the well-known effects of trauma, the study highlights the profound influence of collective trauma on public attitudes toward justice, particularly in sexual offenses. Exposure to extreme violence, during a war characterized by violent crimes creates significant barriers to the acceptance of RJ. While RJ can potentially provide healing avenues for survivors of sexual offenses (Klar-Chalamish & Peleg-Koriat, 2021) the widespread trauma and public anxiety pose significant barriers to its acceptance.

**Disclosure of Interest:**

None Declared

